# Calcitonin Gene-Related Peptide and Substance P As Predictors of Venous Pelvic Pain

**DOI:** 10.32607/20758251-2019-11-4-88-92

**Published:** 2019

**Authors:** S. G. Gavrilov, G. Yu. Vasilieva, I. M. Vasiliev, O. I. Efremova

**Affiliations:** Pirogov Russian National Research Medical University, Moscow, 119049 Russia; Institute of Bio-Medical Problems, Russian Academy of Sciences, Moscow, 123007 Russia

**Keywords:** venous pelvic pain, calcitonin gene-related peptide, substance P

## Abstract

The purpose of this work was to study the contents of calcitonin gene-related peptide (CGRP) and substance P (SP) in the blood plasma of patients with pelvic varicose veins. Thirty women with pelvic varicosities and a reflux blood flow were investigated using duplex ultrasonography. Group 1 included 18 patients with clinical signs of the pelvic congestion syndrome (PCS), including venous pelvic pain (VPP). Group 2 consisted of 12 patients with pelvic varicosities with no clinical signs of PCS. *Group 1. *The score of VPP intensity ranged from 4 to 8; the mean score being 4.84 ± 0.43. The CGRP level in the studied group ranged from 0.39 to 1.01 ng/mL; the SP level ranged from 0.005 to 1.33 ng/mL. *Group 2*. The CGRP values were 0.15–0.32 ng/mL, and the SP range was 0.003–0.3 ng/mL. In this group, the levels of the studied peptides were 3–5 times lower than those for the patients with VPP. *Group 3. *The mean CGRP values were 0.06 ± 0.003 ng/mL, and the mean SP values were 0.03 ± 0.001 ng/mL. These values were considered as the reference parameters; a statistical analysis was performed for them. The correlation analysis revealed a strong relationship between the CGRP and VPP levels (r = 0.82) and a medium correlation between the SP level and pelvic pain in Group 1. The CGRP and SP levels in blood plasma highly correlate with the presence of pelvic venous pain.

## INTRODUCTION

Chronic pelvic pain (CPP) is a highly relevant and challenging problem of modern medicine [1, 2]. According to the World Health Organization (WHO), the prevalence of CPP ranges from 2.4 to 24% of the population, with women of reproductive age being the most predominant group affected [5]. Other data indicate that 3.8% of women suffer from CPP, and that the annual cost of treatment of the disease in Europe amounts to 3.8 billion euro [3, 4]. The pelvic congestion syndrome (PCS) is a cause of CCP in 10–30% of patients with PCS, whereas 10% of the entire female population has pelvic varicose veins and a reflux blood flow and PCS appears in 60% of them [6, 7, 8, 9]. Hansrani et al. (2016) have convincingly proved that there is a relationship between CPP and PCS in women with pelvic vein incompetence [10]. Thus, pelvic venous insufficiency is a serious factor behind the development of CPP. The reasons behind the emergence of venous pelvic pain (VPP) remain unclear, and the available hemodynamic and inflammatory hypotheses cannot fully explain what causes the pain syndrome in some patients and why other patients with identical morphofunctional changes in pelvic veins do not have it [11, 12, 13]. As proved by earlier studies, there is no obvious relationship between the diameter of pelvic veins and the severity of VPP [14, 15]. Meanwhile, the findings obtained by several authors indicate that there might be a relationship between neurogenic inflammation, hyperproduction, and increased activity of vasoactive neuropeptides and the emergence of VPP formation [16, 17, 18, 19].

The objective of this work was to study the levels of calcitonin gene-related peptide (CGRP) and substance P (SP) in the blood plasma of patients with pelvic varicose veins and to determine the degree of correlation between the levels of these algogens and VPP.

## EXPERIMENTAL

**Patients**

Thirty women aged 22–42 years with pelvic varicosities and a pathological reflux blood flow along those veins were enrolled in the study using the results of transabdominal and transvaginal duplex ultrasonography (DUS) of pelvic veins. The study was approved by the Local Ethics Committee of the N.I. Pirogov Russian National Research Medical University and registered at clinicaltrials.gov (NCT03921788). All patients signed a consent form to take part in the study. Group 1 consisted of 18 patients with clinical signs of the pelvic congestion syndrome (PCS), including venous pelvic pain (VPP). The severity of VPP was evaluated using the Visual Analog Scale (VAS). In this group of patients, this parameter ranged from score 4 to 8. Patients in group 2 (12 patients) had pelvic varicose veins but showed no clinical signs of PCS. The inclusion criteria were as follows: women of reproductive age; pelvic vein dilatation and reflux blood flow along parametrial, uterine, and gonadal veins higher than 0.5 s according to the DUS data; the absence of any pathology accompanied by CPP; and signed informed consent form obtained from the patient. The exclusion criteria were the absence of dilated pelvic veins and a reflux blood flow along them during DUS; diseases whose clinical course assumes that patients have CPP and other varieties of the chronic pain syndrome, including migraine. For this purpose, all the patients consulted a gynecologist, an urologist, and a neurologist; they also underwent ultrasonography of internal genitalia and the urinary system.

In addition, 10 healthy subjects without any acute or chronic diseases accompanied by the pain syndrome took part in the study. These subjects had no varicose veins of the pelvis or lower extremities as assessed both visually and according to the DUS data. These patients composed the third (control) group (Group 3).

**Table 1:** Clinical and ultrasonography data (n = 30)

Parameter		Group 1 (n = 18)	Group 2 (n = 12)	Group 3 (n = 10)
Age, years		30.2 ± 2.4	31.6 ± 1.9*	21.3 ± 0.8**
Body mass index (BMI)		23.4 ± 0.8	22 ± 0.6*	20.4 ± 0.3**
Childbearing, n		1–3	1–3	0
Duration of the disease/observation of varicose pelvic veins, years		4.9 ± 1.3	3.3 ± 1.1*	0
Venous pelvic pain, n/%		18/100	0	0
Chronic pain of any other localization, %		0	0	0
Valvular dysfunction	Parametrial veins, n/%	30/100	30/100	0
	Uterine veins, n/%	9/50	5/41.6	0
	Gonadal veins, n/%	4/22.2	3/25	0

* – p > 0.05;

** – p < 0.05.

The results of the clinical and ultrasonography examination are summarized in *Table 1*.

**ELISA (enzyme-linked immunosorbent assay) procedure**

Venous blood was taken from the cubital vein at the same time (8:00–8:30 a.m.) on an empty stomach, in sitting position, and seven days after the end of the last menstruation. The blood was sampled into 4.0 mL vacuum tubes containing K2-EDTA. The blood samples were then centrifuged for 10 min at 3000 rpm. The obtained blood plasma was divided into 1.0 mL aliquots and placed into two Eppendorf tubes. The biological material was immediately frozen and stored at –80°C for subsequent analysis. The levels of calcitonin gene-related peptide (CGRP) and substance P (SP) were determined by competitive enzyme-linked immunosorbent assay (ELISA) using commercial kits (Peninsula laboratories, LLC, Bachem Group, USA). The reference and test samples were analyzed in doublets. Protocol no. 5, recommended by the manufacturer (incubation at 4°C for 14–16 h (overnight)), was used. The absorbance was measured on a Stat Fax 2100 immunoenzymatic analyzer (microplate photometer, Awareness Technology Inc., USA) in standard 96-well plates at a wavelength of 450 nm. Concentrations of neuropeptides were calculated using the Cobas EIA recalibration software (F. Hoffmann – La Roche Ltd, Switzerland).

**Statistical analysis**

The statistical analysis was performed using the Microsoft Excel and Statistica 6.0 software and the medstatistic.ru statistical online calculator. The arithmetic mean (M) and standard deviation (σ) were calculated. The data are presented as absolute and relative values. The differences were considered statistically significant at *p * < 0.05. Correlation regression analysis (r) and calculation of the relative risk (RR) were used to evaluate the relationships between the clinical and laboratory parameters.

## RESULTS AND DISCUSSION

**Duplex ultrasonography data**

The transabdominal and transvaginal DUS data indicated that there were no significant distinctions in the incidence rate of valvular insufficiency of pelvic veins in the two groups of patients. No symptoms of pelvic congestion syndrome (PCS) were observed in Group 2 patients in spite of the pathological reflux blood flow along the gonadal (25%) and uterine (41.6%) veins. The diameter of intrapelvic veins was ignored, because there was no significant correlation with the presence and severity of VPP as confirmed by previous studies [14,15]. Statistically significant intergroup differences were observed for the laboratory results.

## ELISA DATA

**Group 1**

Among the patients in this group, the severity of VPP ranged from score 4 to 8; the mean score was 4.84 ± 0.43. The CGRP level in the studied group ranged from 0.39 to 1.01 ng/mL (mean, 0.71 ± 0.11 ng/mL); the SP level ranged from 0.005 to 1.33 ng/mL (mean, 0.42 ± 0.18 ng/mL). The CGRP levels lay in the range of 0.69–1.01 ng/mL, the SP level, from 0.006 to 1.45 ng/mL. In two patients with maximum pain severity (score 8), a combination of increased levels of neuropeptides was revealed: in one patient, the CGRP and SP levels were 0.69 and 1.33 ng/mL, respectively; in another patient, these values were 1.01 and 1.45 ng/mL, respectively. The simultaneous increase in the production of these proteins probably contributes to the aggravation of the pain syndrome. In six patients, pelvic pain with a severity score = 4 was accompanied by a less significant increase in the levels of CGRP (0.39–0.51 ng/mL) and SP (0.005–0.38 ng/mL). *Figure* shows the clinical and laboratory parallels between the severity of VPP and the levels of neurotransmitters under study.

**Fig. 1:**
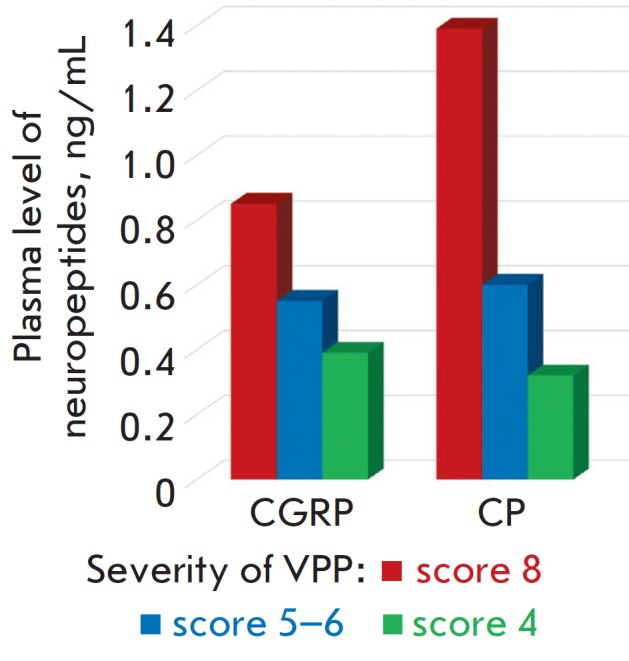
The CGRP and SP levels and severity of venous pelvic pain in group 1 patients

In contrast to the CGRP level, the plasma levels of SP varied widely, from normal values to a significant increase of up to 1.45 ng/mL. The cause of this phenomenon will be investigated in further studies.

**Group 2**

No VPP was observed in Group 2 patients. The CGRP levels were 0.15–0.32 ng/mL (mean, 0.26 ± 0.02 ng/mL); the SP levels were 0.003–0.3 ng/mL (mean, 0.15 ± 0.06). In this group, the levels of the studied neuropeptides were 3–5 times lower than those in patients with VPP. No correlations between the GGRP and SR levels were revealed in patients without pelvic pain.

**Group 3**

No signs of chronic pain syndrome of any localization were observed in healthy subjects. The mean CGRP and SP levels were 0.06 ± 0.003 and 0.03 ± 0.001 ng/mL, respectively. These levels were considered as the reference values and were used for the statistical analysis.

The correlation analysis showed a strong relationship between the CGRP and VPP levels (r = 0.82) and a medium relationship between the SP level and the pelvic pain severity in Group 1 patients. The calculated relative risk (RR) of developing VPP with increasing CGRP level in Group 1 is 19-fold higher than that in Group 2 (*RR *= 19.19; 95% CI: 2.78–132.35) and indicates that there is a direct relationship between VPP severity and the CGRP level. No such evident correlations were revealed for Group 2.

*Table 2* lists the VPP severity and the CGRP and SP levels in the studied groups.

**Table 2:** Severity of VPP and plasma levels of CGRP and SP in the study groups

Parameter	Group 1 (n = 18)	Group 2 (n = 12)	p*	Group 3 (n = 10)	p**
VPP, score	4.84 ± 0.43	0	-	0	-
CGRP level, ng/mL	0.71 ± 0.11	0.26 ± 0.02	p = 0.0004	0.06 ± 0.003	p = 0.0001
SP level, ng/mL	0.42 ± 0.18	0.15 ± 0.06	p = 0.166	0.03 ± 0.001	p = 0.05

* – Groups 1 and 2 were compared;

** – Groups 2 and 3 were compared.

Significant differences in the plasma levels of CGRP were revealed for Groups 1 and 2. The differences in the plasma level of SP for these two groups are statistically insignificant, but this parameter apparently tends to increase in patients with VPP. The CGRP and SP levels in Group 3 are statistically significantly lower than those in Group 2, which probably indicates that the mere existence of varicose veins can be accompanied by an increase in the levels of these neuropeptides regardless of whether or not patients display the pain syndrome.

Back in 1985, J.A. Fisher and W. Born observed pronounced cardiovascular effects for CGRP injected intravenously (vasodilatation, hypotension, positive chronotropic and inotropic effects on the heart) [20]. The maximum efficacy of CGRP was observed at the microcirculation level (its vasodilatory activity was tenfold higher than that of prostaglandins). CGRP is abundant in the peripheral and central nervous system; its receptors are expressed in the pain pathways and usually colocalize with other neuropeptides, including substance P [21]. Receptors to CGRP and SP were also observed in the pelvic veins of women [17, 22]. Stones et al. (1995) detected SP in endothelial cells of the ovarian vein and proved that it is involved in the regulation of the vascular tone of this vessel [22]. They suggested that the disruption of venous outflow in women with PCS increases the elimination of CP, and that the hypersensitivity of receptors to this neuropeptide causes the pain syndrome. The synergistic effect of substance P and CGRP on the venous tone may play a significant role in the occurrence of venous pelvic pain. The number and sensitivity of receptors to these neurotransmitters probably determine whether or not patients with PCS will develop venous pelvic pain. Stones et al. found that intravenous injection of CGRP to patients with PCS leads to a high SP level in endothelial cells of ovarian veins and aggravation of pelvic pain. This proved a compelling argument for studying the influence of these neurotransmitters on the development of VPP in patients with PCS.

The reported results of the study of the plasma levels of CGRP and SP in groups of patients with pelvic varicosities accompanied by a reflux blood flow indicate that there is a tight correlation between the level of these neuropeptides and pelvic pain. To a certain extent, this fact indirectly confirms the theory of a vein-specific inflammation that emerges during varicose vein transformation and is accompanied by vein wall hypoxia, which should be regarded as a damaging factor contributing to neurogenic inflammation in the vein wall, enhanced synthesis of neuropeptide algogens, and development of the pain syndrome.

Today, the reference CGRP and SP levels in healthy people are unknown. The available data is contradictory: some of the data indicate that the plasma of healthy people does not contain these substances. Meanwhile, other data strongly indicate that the normal CGRP level ranges from 2 to 36 pmol/L and that of SP does not exceed 0.1–0.19 ng/mL [23, 24, 25]. Our study demonstrates that the CGRP and SP levels in healthy female subjects do not exceed 0.06 ± 0.003 and 0.03 ± 0.001 ng/mL, respectively. However, the distinctions in the test systems used by independent authors should be taken into account. In our work, we report on the preliminary results of a study that will be continued until the necessary power and representativeness are achieved. Meanwhile, the obtained data indicate that the chosen scientific research is quite promising.

It should be noted that CGRP and SP are only two vasoactive neuropeptides whose levels were studied in patients with venous pelvic pain. However, the development of pain in patients with PCS involves the activation of the entire range of neurotransmitters and algogens (neurokinin A, endothelin, prostaglandins, nitric oxide, interleukin-1, tumor necrosis factor-α, etc.). In particular, Agu et al. (2002) and Yang et al. (2008) showed that decreased expression of endothelin-1 (ET-1), in combination with a decreased number of endothelin-B receptors, is a factor responsible for a reduction of the vasoconstrictor activity of veins and their varicose transformation [26, 27]. Pietrzycka et al. (2015) found that therapy with a micronized purified flavonoid fraction in female patients with a chronic venous disease (CVD) is accompanied by an increase in ET-1 levels, while the level of tumor necrosis factor-α (TNF-α) decreases, which indirectly indicates that ET-1 participates in the regulation of the venous tone in patients with CVD [28]. These data suggest that further research into the neurobiological aspects of venous pelvic pain is needed, which could allow one to evaluate the effect of other protein derivatives on the pathological processes taking place in the vein wall.

## CONCLUSION

The plasma levels of CGRP and SP strongly correlate with pelvic venous pain. These neuropeptides probably play a substantial role in the development of the pain syndrome in patients with the pelvic venous congestion syndrome. The high levels of CGRP and SP in patients with VPP resistant to conventional phlebotropic therapy can be an indication towards administering medications that block these neurotransmitters to treat such patients.

## Abbreviations

VPP: venous pelvic pain
PCS: pelvic congestion syndrome
CGRP: calcitonin gene-related peptide
SP: substance P
